# Long-Term In Vivo Evaluation of Chitosan Nerve Guide Properties with respect to Two Different Sterilization Methods

**DOI:** 10.1155/2018/6982738

**Published:** 2018-06-04

**Authors:** Maria Stößel, Jennifer Metzen, Vivien M. Wildhagen, Olaf Helmecke, Lena Rehra, Thomas Freier, Kirsten Haastert-Talini

**Affiliations:** ^1^Institute of Neuroanatomy and Cell Biology, Hannover Medical School, Carl-Neuberg-Str. 1, 30625 Hannover, Germany; ^2^Center for Systems Neuroscience (ZSN), 30559 Hannover, Germany; ^3^Medovent GmbH, Friedrich-Koenig-Str. 3, 55129 Mainz, Germany

## Abstract

Severe peripheral nerve injuries are reconstructed either with autologous nerve grafts (gold standard) or alternatively with clinically approved artificial nerve guides. The most common method used to sterilize these medical products is ethylene oxide gassing (EO). However, this method has several disadvantages. An alternative, which has been barely studied so far, represents beta irradiation (*β*). In previous studies, we developed an artificial nerve guide made of chitosan (chitosan nerve guide, CNG), a biomaterial that is known to potentially retain toxic residues upon EO sterilization. Therefore, we analyzed the long-term regeneration-supporting and mechanical properties of CNGs upon their sterilization with EO or *β* and their following application in unilateral repair of 12 mm gaps of the rat sciatic nerve. Over a period of 76 weeks, we serially evaluated the recovery of motor functions, the possible emergence of an inflammation in the surrounding connective tissue, the regrowth of axons into the distal nerve, and possible changes in the material properties. Our first long-term evaluation did not reveal significant differences between both sterilization methods. Thus, *β* is as appropriate as commonly used EO for sterilization of CNGs; however, it may slightly increase the stiffness of the biomaterial over time.

## 1. Introduction

Chitosan, a random copolymer consisting of D-glucosamine units (C_6_H_11_NO_4_)_n_) and N-acetyl-D-glucosamine units (C_8_H_13_NO_5_)_n_), is a highly biocompatible, natural-derived heteropolymer that is widely used as scaffold-material in different biomedical applications [1–3]. In recent years, we contributed to the development of a novel nerve guidance conduit made of chitosan for the purpose of peripheral nerve reconstruction after severe transection injuries. In different experimental studies, these chitosan nerve guides (CNGs) have demonstrated very good properties to support critical gap lengths (15 mm) rat sciatic nerve regeneration in acute as well as in clinically relevant delayed repair approaches [4–8]. Over time, different mechanical properties were monitored, e.g., the flexibility, the compression resistance, and the degradation rates of the CNGs, to finally deliver the ideal implant for clinical application [6, 7]. Based on the overall promising results of our studies, CNGs entered the market in 2014 (as Reaxon® Nerve Guide by Medovent GmbH, Germany).

Biomedical products need to undergo tightly controlled sterilization processes before clinical application. Numerous methods exist to achieve sterilization of biocompatible materials [9–12], with regard to chitosan, the biomaterial this work is focused on, ethylene oxide gassing (EO) is the second most commonly reported sterilization method besides the usage of ethanol aqueous solutions that are only suitable for small-scale applications [13]. Advantages of EO are a reliable penetration at low temperatures and the proven compatibility with a variety of materials [13–15]. But EO is also reported to be highly carcinogen and explosive; thus its application has to follow strict rules [13–15]. Furthermore, toxic residues might remain at the treated materials' surfaces by irreversible alkylation of highly reactive functional groups such as the amine groups of the chitosan structure [13–15]. In this context, variations in the degradation rates of collagen have been reported [14].

An alternative to EO represents the exposure of biomedical products to ionizing sources in which gamma irradiation is commonly used for implant materials [14, 16]. Besides its radioactivity, the main downside reported for this method in the sterilization of chitosan is a reduction of its mechanical strength caused by considerable chain scissions [13, 16]. The sterilization of natural-based, biomedical materials by beta irradiation (*β*) is another alternative. And although barely mentioned in the literature so far, it is reported to only induce negligible changes to material properties [14, 17].

In the current study, we regularly evaluated the mechanical properties of CNGs, which underwent either *β* or EO sterilization and in parallel monitored functional recovery over a period of 76 weeks in a long-term experimental approach of reconstructing 12 mm rat sciatic nerve gaps.

## 2. Materials and Methods

### 2.1. Experimental Design

We examined the long-term influence of two different sterilization methods, i.e., ethylene oxide gassing (EO) and beta irradiation (*β*), on the material properties of chitosan nerve guides (CNGs) with a degree of acetylation (DA) of 5%. Therefore, 48 female Lewis rats (EO: n=24; *β*: n=24) were subjected to unilateral sciatic nerve injury and repair (nerve gaps: 12 mm). Starting from 16 weeks after surgery, functional recovery was monitored every 20 weeks by transcutaneous electrodiagnostic measurements for the following 60 weeks. At every time point (16, 36, 56, and 76 weeks), six animals per group were sacrificed for interim evaluations of the tube properties, the connective tissue, which had formed on the outside of the CNGs, and nerve morphometry distal to the implant. Age-related death reduced the number of animals within the EO group at 42, 61, and 65 weeks after surgery without any correlation to the applied sterilization method.

### 2.2. Manufacturing and Sterilization of Chitosan Nerve Guides

The chitosan material was derived from Pandalus borealis shrimp shells and processed to medical grade by Chitinor AS (Tromsø, Norway). CNGs were produced according to ISO 13485 requirements in a length of 16 mm, with an inner diameter of 2.1 mm, a wall thickness of 0.3 mm (dry condition) and of 0.7 mm in wet condition (with preserved inner diameter), and a DA of 5% by Medovent GmbH (Mainz, Germany). Before application, CNGs were either EO sterilized by HA2 Medizintechnik GmbH (Halberstadt, Germany) or *β* sterilized (11 kGy, 10 MeV) by BGS Beta-Gamma-Service GmbH & Co. KG (Wiehl, Germany).

### 2.3. Animals and Surgical Procedure

Animal experiments have been approved by the animal care committee of Lower-Saxony, Germany (approval code: 33.12-42502-04-15/1761; approval date: April 10th, 2015). 48 female Lewis rats (initial average weight: 173.1±1.3 g) were housed in groups of 4 (room temperature 22.2°C; humidity 55.5%; light/dark cycle of 14 h/10 h). Amytriptiline hydrochloride (13.5 mg/kg/day, Amitriptylin-neuraxpharm®, Neuraxpharm Arzneimittel GmbH, Germany) was added to the drinking water to prevent automutilation starting two weeks before surgery [18]. Food and water were provided ad libitum. The animals' health state was controlled every 2-3 days.

Aseptic conditions and adequate anesthesia and analgesia were applied for surgery and electrodiagnostic recordings. Deep anesthesia was induced by intraperitoneal injection of Chloral hydrate (370 mg/kg, Sigma-Aldrich Chemie GmbH, Germany). Sufficient analgesia was achieved by applying bupivacaine (0.25%, Carbostesin®, AstraZeneca GmbH, Germany) and lidocaine (2%, Xylocain®, AstraZeneca GmbH, Germany) locally on the exposed sciatic nerves 5 min prior to nerve transection. Further pain relief was achieved by subcutaneous injection of butorphanol (0.5 mg/kg, Torbugesic®, Pfizer GmbH, Germany) at the day of surgery/ electrodiagnostic measurements and the two following days.

CNGs were, in accordance with the manufacturers' advice for clinical use, rinsed in 0.9% sodium chloride solution (NaCl 0.9%, B. Braun Melsungen GmbH, Germany) for at least 20 min and surgeries performed as described before [4–6]. Briefly, the left sciatic nerves were exposed at mid-thigh level and transected with a microscissor at 2 mm distal to the aponeurosis of the gluteus muscle. The nerves had a diameter of appr. 1 mm and after transection the separated nerve ends naturally retracted for about 5 mm. A 6 mm nerve piece was removed from the distal nerve end. The free nerve ends were inserted in the respective CNGs (EO or *β* sterilized, lengths: 16 mm) with 2 mm overlaps at each side and fixed using one epineural stitch at each side of the CNGs (9-0 Ethilon, EH7981G, Ethicon, Scotland) resulting in 12 mm nerve gaps. All measures given above were controlled and proven using a strip of sterile scale paper (mm scale) in each of the operated animals. Wound closure was performed with 3-4 resorbable sutures for the femoral biceps muscle (3-0 Polysorb, UL-215, Syneture, USA) and 3-4 nonresorbable mattress sutures (4-0 Ethilon™II, EH7791H, Ethicon, Scotland) for the skin.

### 2.4. Assessment of Functional Motor Recovery with Transcutaneous Electrodiagnostic Recordings

Functional recovery over time was monitored with transcutaneous electrodiagnostic measurements at 16, 36, 56, and 76 weeks after surgery as described earlier in deeply anaesthetized animals placed in prone-position on a heating pad [4–6]. The investigator was blinded to the sterilization methods used on the implanted nerve guides.

Transcutaneous monopolar needle electrodes connected to a Dantec® Keypoint® Focus device (Natus Europe GmbH, Germany) delivered single electrical impulses with an increasing intensity (100*μ*s duration with supramaximal intensity at 1Hz frequency) to the sciatic nerve either proximal (sciatic notch) or distal to the implantation site (fossa popliteal). Evoked compound muscle action potentials (CMAPs) of the tibialis anterior (TA) and plantar (PL) muscles were recorded by means of monopolar needles (28G; dermal EEG electrodes) inserted across the skin on the muscle belly. Therefore, the recording needles were placed guided by anatomical landmarks to secure the same placement on all animals. The active recording electrode was placed subcutaneously at the first third of the distance between knee and ankle on the TA muscle and at the third metatarsal space for PL muscle recordings. The reference needle electrode was inserted at the distal phalange of the fourth toe. A ground needle electrode was inserted in the skin at the dorsum of the foot or at the knee. Recordings from the contralateral healthy sides served as control. For more details and an illustration of needle placement the reader is kindly referred to our previous work [6] and informed that we followed a standard procedure in the field [19]. By calculating ratios of CMAP amplitudes (baseline to negative peak of the M-wave) and nerve conduction velocities (NCVs) between the reconstructed and uninjured sides (lesioned side divided by nonlesioned side), the degree of functional motor reinnervation was determined. Additionally, the axon loss was assessed as described earlier [20]. In cases, where no evoked CMAPs were recordable, CMAP amplitude and NCV ratios were set to 0 while the axon loss was set to 100%. However, all results were included into final statistical analysis.

### 2.5. Immunohistochemistry

At 16, 36, 56, and 76 weeks after surgery, six animals of each group were sacrificed. For ethical reasons and to limit the period the animals had to suffer from suboptimal recovery of nerve function, the six animals per group showing the comparably worst results in the electrodiagnostic measurements at each related time point were chosen for interim evaluations. Upon harvest of the transplants, each specimen was divided into the connective tissue that was surrounding the CNG (immunohistological evaluation), the distal nerve segment (histomorphometrical analysis), and the separated CNG (analysis of material properties). The investigator was blinded to the sterilization methods used on the implanted nerve guides.

At first, the connective tissue samples were macroscopically classified concerning their optical density (three categories: mainly translucent, partly translucent/partly opaque, and mainly opaque). Then, the connective tissue was folded, with the CNG contact surfaces facing each other, and fixed overnight at 4°C in 4% paraformaldehyde (PFA; Sigma-Aldrich Chemie GmbH, Germany) diluted in phosphate buffered saline (PBS; Dulbecco, Biochrom GmbH, Germany). Afterwards, the samples were paraffin-embedded and series of 40 blind-coded cross sections (thickness: 7 *μ*m) starting from the distal end of the connective tissue prepared. As previously described [6], sections were first stained with hematoxylin and eosin (HE) and mounted using Mowiol (Calbiochem GmbH, Germany). For further analysis, representative photomicrographs were processed as multiple image alignments (MIAs) with the Stereo Investigator software version 11.07 (MBF Bioscience, USA) using a BX51 microscope (Olympus, Germany) that was expanded with a joystick controlled microscope stage (MBF Bioscience, USA). In 4 randomly selected sections the connective tissue cross-sectional areas and thicknesses were measured with the cellSens Entry software version 1.5 (Olympus, Germany), and the number of multinucleated giant cells and chitosan fragments was determined. Consecutive cross sections were immunohistologically stained for activated macrophages (ED1) in order to assess the degree of a possible foreign body response. Upon blocking in 5% rabbit serum (Sigma-Aldrich GmbH, Germany) diluted in PBS, sections were stained with primary mouse anti-ED1 antibody (1:1000 diluted in blocking solution; MCA 275R Serotec, UK) overnight at 4°C, washed thrice in PBS, and stained with Alexa 555-conjugated secondary goat anti-mouse antibody (1:1000 diluted in blocking solution; A21422, Invitrogen, Germany) for 1 h at room temperature (RT). After washing in PBS again, 4',6'-diamidino-2-phenylindole (DAPI; 1:2000 diluted in PBS; Sigma-Aldrich GmbH, Germany) was applied for nuclear counterstaining (2 min at RT), before mounting with Mowiol. Representative photomicrographs were taken at 20x magnification with the cellSens Dimension software version 1.15 (Olympus, Germany) using a BX60 microscope (Olympus, Germany). In each of two stained connective tissue cross sections (distance between both: 140 *μ*m), 4 randomly selected areas were evaluated for the number of ED1-immunopositive signals per mm^2^ using the ImageJ software version 1.48 (National Institutes of Health, USA). DAPI staining served to clearly identify ED1-immunopositive cells.

### 2.6. Histomorphometry

Nerve segments distal to the CNGs and distal portions of control healthy nerves (n=3 at each 16, 36, 56, and 76 weeks) were histomorphometrically analyzed as described before [21]. During our analysis we have considered internationally accepted procedures as described in key literature [22–25].

Briefly, nerve samples were first fixed in Karnovsky solution (2% PFA and 2.5% glutaraldehyde in 0.2 M sodium cacodylate buffer, pH 7.3) for 24 h and rinsed in 0.1 M sodium cacodylate buffer containing 7.5% sucrose and postfixed in 1% osmium tetroxide for 1.5 h. Myelin sheaths were stained with 1% potassium dichromate (for 24 h), 25% ethanol (for 24 h), and hematoxylin (0.5% in 70% ethanol, for 24 h). Dehydrated nerve ends were Epon-embedded and cut in semithin cross sections (thickness: 1 *μ*m). Toluidine blue staining was applied to enhance myelin sheath staining before sample sections were mounted using Mowiol.

As previously described [21], two sections from three randomly selected animals per group and time point (n=3; if no reinnervation was detectable during electrodiagnostic evaluation, the n-count was decreased accordingly) were examined at light microscopic level with the Stereo Investigator software version 11.04 (MBF Bioscience, USA) using a BX50 microscope (Olympus GmbH, Germany) expanded with a prior controller (MBF Bioscience, USA). Besides the evaluation of the total cross-sectional area at 20x magnification, the total fiber number and the mean nerve fiber density were assessed at 100x magnification using a two-dimensional procedure as described before [21] (optical fractionator; grid size: 150x150*μ*m^2^; counting frame size: 30x30*μ*m^2^; counting of “fiber tops”). For the evaluation of regeneration-related size parameters (axon and fiber diameters, myelin thicknesses, and* g*-ratios), photomicrographs of four randomly selected areas of each section were taken at 100x magnification and evaluated with the* g*-ratio plug-in (http://gratio.efil.de/) in ImageJ software version 1.48 (National Institutes of Health, USA). Therefore, 80 axons were evaluated for each animal following the theoretical assumption that they were circular. The investigator was blinded to the sterilization methods used on the implanted nerve guides.

### 2.7. Analysis of the Explanted Chitosan Nerve Guides

Upon harvest of the CNGs, their macroscopical appearance was analyzed concerning visible fissures or fragmentation prior to evaluation of their mechanical properties. After freshly produced or explanted CNGs have been placed in PBS for 12 h at 37°C, their flexibility was assessed by insertion of a nylon thread (diameter: 0.1 mm) into the lumen of the soaked CNGs in order to pull the CNGs around cylindrical rods of different diameters (diameter range: 0.5-6.0 mm). The minimum curvature radius until lumen closure determined the maximum bendability of the respectively tested CNG.

Additionally, the compression-stability of the CNGs was analyzed using a mechanical test device (type Z3, Thümler GmbH, Germany). At a crosshead speed of 1 mm/min, the applied force was measured when a displacement of 60% perpendicular to the longitudinal axis of the CNGs could be observed.

### 2.8. Statistics

All results obtained in this study, except for the connective tissue optical density classification, were subjected to statistical analysis using the GraphPad Prism software version 6.05 (GraphPad Software, USA). A p-value of p<0.05 was set as level of significance. A chi-square test was used for statistical analysis of the animal numbers displaying positive signals during electrophysiology. In this evaluation, data was presented as percentages. All other results were analyzed using a two-way ANOVA (Analysis of Variance) followed by Tukey's multiple comparison. These results were displayed as median±range. N-values are given in the respective figure legends.

## 3. Results and Discussion

### 3.1. Assessment of Functional Motor Recovery with Transcutaneous Electrodiagnostic Recordings

**Figure 1:**
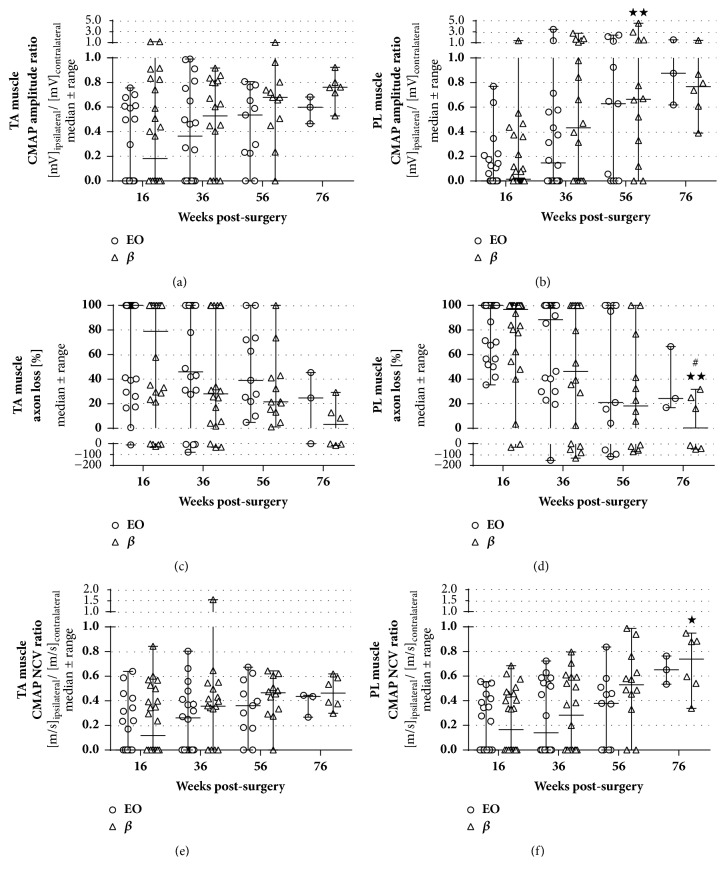
**Electrodiagnostic recordings depicting functional motor recovery at 16, 36, 56, and 76 weeks after surgery.** Evoked compound action muscle potentials (CMAPs) were recorded from the anterior tibial (TA; (**a**), (**c**), (**e**)) and plantar muscles (PL; (**b**), (**d**), (**f**)) to evaluate amplitude ratios (**a**,** b**), axon loss (**c**,** d**), and nerve conduction velocity (NCV) ratios (**e**,** f**). Both groups, i.e., EO and *β* (both: n=24 at 16 weeks, n=18 at 36 weeks; EO: n=11 at 56 weeks, n=3 at 76 weeks; *β*: n=12 at 56 weeks, n=6 at 76 weeks) improved over time without significant differences between the groups. Two-way ANOVA followed by Tukey's multiple comparison was used for statistical analysis of the results (*∗*p<0.05, *∗∗*p<0.01 versus 16 weeks within the same group; ^#^p<0.05 versus 36 weeks within the same group). Results are displayed as median±range.

During the whole long-term study, no significant differences between the usage of nerve guides that were sterilized by either ethylene oxide gassing (EO) or beta irradiation (*β*) were detected in the electrodiagnostic measurements (Table 1, Figure 1). At 16 weeks after surgery, approximately half of all animals demonstrated evocable compound muscle action potentials (CMAPs) in the anterior tibial (TA) and plantar (PL) muscles, with the number slightly increasing in the *β* group (Table 1). Within the next 60 weeks, there was an overall increase of motor recovery in both groups (Table 1). Most of the animals did, however, not reach the degree of motor function observed in the contralateral healthy side (CMAP amplitude or NCV ratio of 1, axon loss of 0%). The progression in motor recovery could be observed in the evaluation of CMAP amplitude ratios (Figures 1(a) and 1(b)), accompanied by decreasing axon loss (Figures 1(c) and 1(d)), and increasing CMAP nerve conduction velocity (NCV) ratios (Figures 1(e) and 1(f)). However, it needs to be reminded that after each evaluation six animals of each group with the least improvement were sacrificed for the evaluation of material-dependent long-term effects at the implantation site.

### 3.2. Evaluation of the Connective Tissue Surrounding the Nerve Guides (Immunohistochemistry)

**Figure 2:**
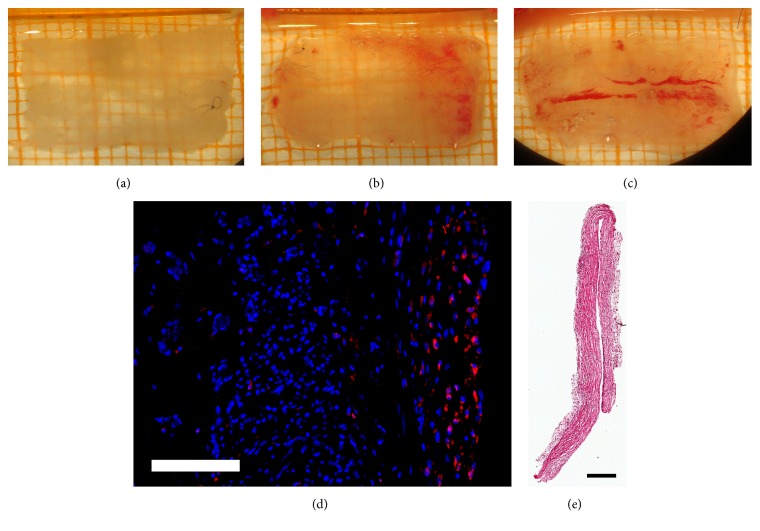
**Photomicrographs illustrating the optical density of the connective tissue as well as examples of ED1/DAPI- and H&E stained sections.** Optical density of the explanted connective tissue was classified as (**a**) translucent, (**b**) translucent-opaque, and (**c**) opaque. (**d**) Example photomicrograph showing DAPI-stained nuclei in blue and ED1-immunopositive cells in red; scale bar 100 *μ*m. (**e**) Example photomicrograph of a H&E stained section through the analyzed connective tissue; scale bar 250*μ*m.

To examine a possible foreign body reaction induced by the different sterilization methods over time, we comprehensively analyzed different characteristics of the connective tissue that had formed around the CNGs in six animals per group and time point.  Figure 2 shows photomicrographs illustrating the macroscopic parameters analyzed and a characteristic H&E stained section as well as a sample photomicrograph of the immunohistochemical staining performed.

**Figure 3:**
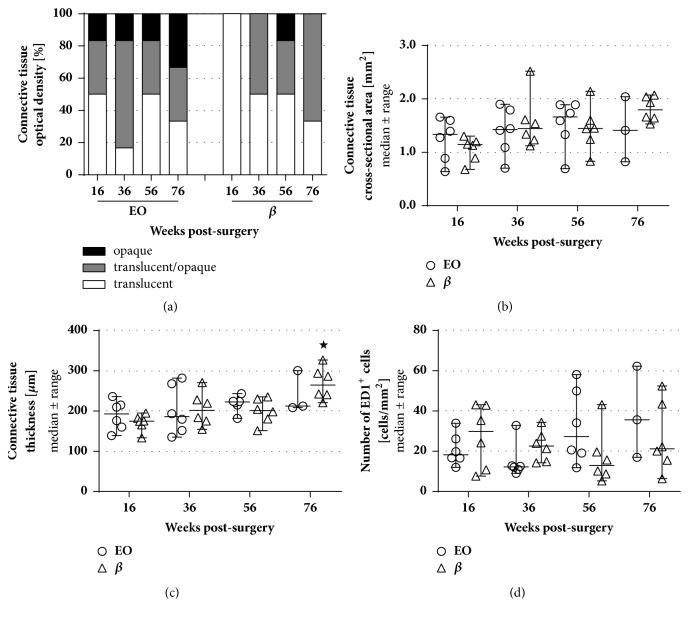
**Analysis of the connective tissue properties related to an immunoreaction including the macroscopically evaluable optical densities, the cross-sectional areas and thicknesses, and the numbers of activated macrophages.** (**a**) The unfolded connective tissues were classified in three categories (mostly translucent, partly translucent and opaque, and predominantly opaque). In the EO group, opaque tissue was found at every time point whereas specimen of the *β* group were predominantly translucent and partly opaque. The classification is presented as percentages per group (0-100%) and was not subjected to statistical analysis. Concerning the evaluated cross-sectional areas (**b**) and thicknesses (**c**) as well as the numbers of ED1-immunopositive (ED1^+^) cells (**d**), no significant differences were found between both groups (both groups: n=6 at 16, 36, 56 weeks; EO: n=3 at 76 weeks; *β*: n=6 at 76 weeks). Steady values in cross-sectional areas and thicknesses combined with low numbers of activated macrophages do not indicate an increased immunoreaction. Two-way ANOVA followed by Tukey's multiple comparison was used for statistical analysis of the results (*∗*p<0.05 versus 16 weeks within the same group). Results are displayed as median±range.

As a first parameter related to a potentially increased immunoreaction, the optical densities of the connective tissue were macroscopically assessed (Figure 3(a)). Overall, the connective tissues that had formed around EO sterilized tubes led to an increased optical density with higher amounts of opaque and lower amounts of translucent samples compared to the *β* group. But histological evaluation revealed no significant differences between both groups for the cross-sectional areas and thicknesses of the analyzed tissue at any time (Figures 3(b) and 3(c)). However, a significant increase in the connective tissue thicknesses could be observed in the *β* group between 16 and 76 weeks after surgery whereas the connective tissue cross-sectional areas remained at a constant level within both groups over time. In the same sections, the number of detectable macrophage giant cells was evaluated as earlier described [6]. Only in HE-stained cross sections of two animals (EO group at 16 weeks and *β* group at 36 weeks after surgery), macrophage giant cell formations were detected. Furthermore, the number of ED1-immunopositive cells indicating activated macrophages was evaluated in immunohistologically stained cross sections of the connective tissue samples (Figure 3(d)). Again, results were similar between both groups and remained stable until the end of the study.

### 3.3. Evaluation of the Distal Nerve End (Histomorphometry)

**Figure 4:**
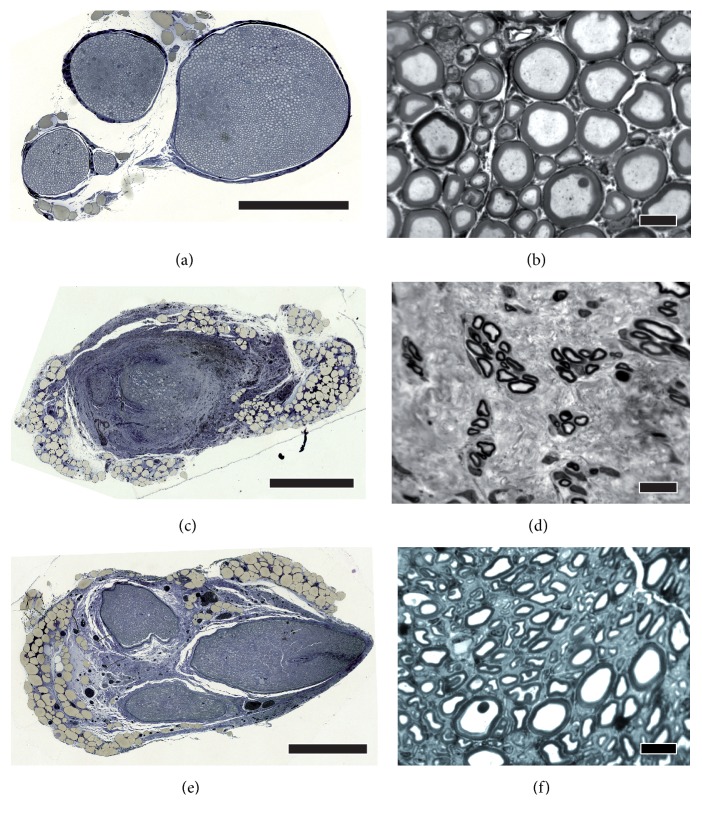
**Photomicrographs illustrating tissue section histomorphometrically analyzed.** (**a**,** b**) Cross-section through an age- and location-matched health nerve, (**c**,** d**) nerve harvested 16 weeks after reconstruction, and (**e**,**f**) nerve harvested 76 weeks after reconstruction. (**a**,** c**,** e**) Scale bars: 500 *μ*m; (**b**,**d**,**f**) scale bars 10 *μ*m.

**Figure 5:**
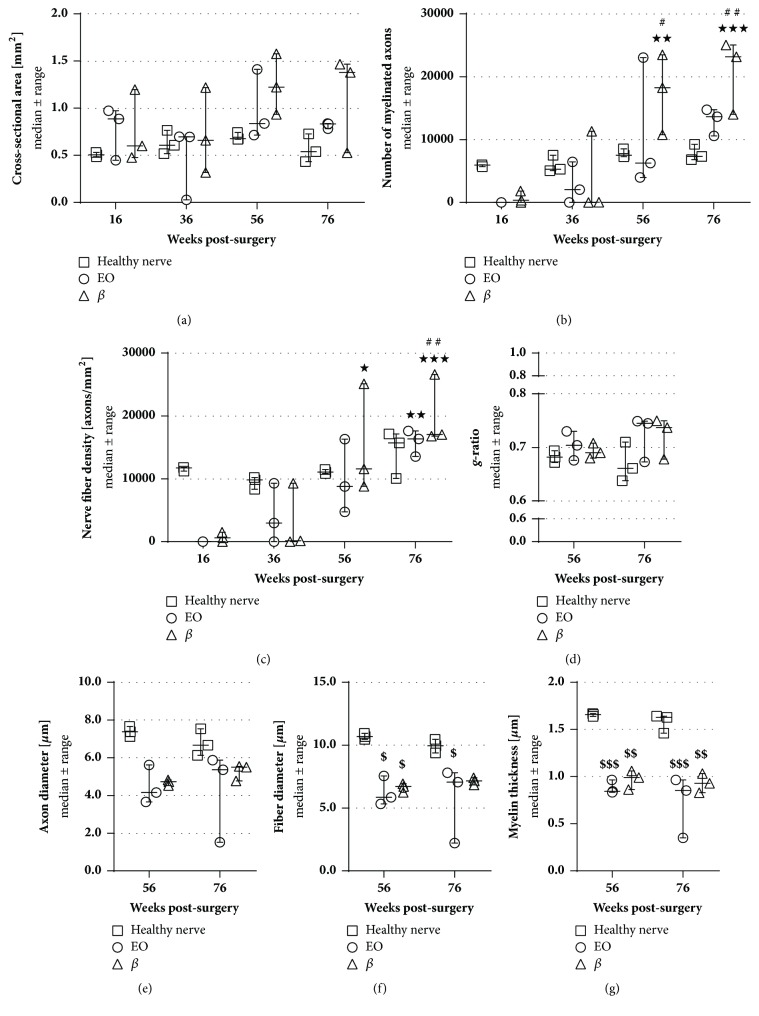
**Histomorphometrical analysis of distal nerve ends compared to healthy nerve samples of age-correlated animals.** In total cross sections, the cross-sectional area (**a**), the number of myelinated nerve fibers (**b**), and the nerve fiber density (**c**) have been evaluated at every time point (16, 36, 56, and 76 weeks; n=3). No significant differences were found between both groups while both significantly increased in nerve fiber density. Analysis of regeneration-related size parameters, i.e.,* g*-ratio (**d**), axon diameter (**e**), fiber diameter (**f**), and myelin thickness (**g**), was performed at 56 and 76 weeks after surgery and revealed significant smaller fiber diameters and myelin thicknesses in both groups compared to healthy nerve specimen (n=3). Two-way ANOVA followed by Tukey's multiple comparison was used for statistical analysis of the results (*∗*p<0.05, *∗∗*p<0.01, *∗∗∗*p<0.001 versus 16 weeks within the same group; ^#^p<0.05, ^##^p<0.01 versus 36 weeks within the same group; ^$^p<0.05, ^$$^p<0.01, ^$$$^p<0.001 versus healthy nerve at the same time). Results are displayed as median±range.

Nerve ends distal to the CNGs were histomorphometrically analyzed in comparison to healthy age- and location-matched contralateral nerve samples.  Figure 4 demonstrates the appearance of the analyzed tissue sections taken from healthy nerves and experimental nerves 16 weeks and 76 weeks after reconstruction. Concerning the cross-sectional areas (Figure 5(a)), the number of myelinated nerve fibers (Figure 5(b)), and the resulting nerve fiber densities (Figure 5(c)), no significant differences could be found neither between both groups nor compared to healthy nerve samples. At 56 and 76 weeks after surgery, nerve ends of the *β* group revealed higher numbers of nerve fibers compared to earlier time points. By the end of the study, both groups led to a significantly increased nerve fiber density compared to the beginning of the study, e.g., at 16 weeks after surgery, when none of the sacrificed samples displayed any regrown axons in the distal nerve ends. Furthermore, different size-related parameters were assessed, i.e., the* g*-ratios (Figure 5(d)), the axon (Figure 5(e)) and fiber diameters (Figure 5(f)), and the myelin thicknesses (Figure 5(g)). Since no axons were found in the distal nerve ends of both groups at 16 and 36 weeks after surgery, only later time points were included in this analysis. Both groups revealed significantly lower values compared to the healthy samples at 56 and 76 weeks after surgery for fiber diameters and myelin thicknesses, while no significant differences were detected for the* g*-ratio.

### 3.4. Analysis of the Explanted Chitosan Nerve Guides

**Figure 6:**
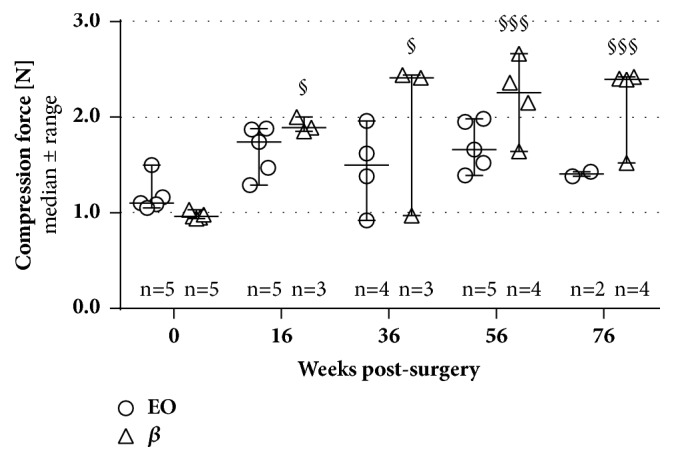
**Evaluation of the compression-stability of both tube types presurgically and at all time point postsurgically.** Tubes that were presurgically sterilized by *β* irradiation showed a significant increase in their stiffness over implantation time; i.e., a higher force was necessary to compress these tubes by the same degree as EO-treated tubes (respective n-values are given in the graph; variations in n-counts occurred due to an increased brittleness of both tube types). Two-way ANOVA followed by Tukey's multiple comparison was used for statistical analysis of the results (^§^p<0.05, ^§§^p<0.01, ^§§§^p<0.001 versus 0 weeks, i.e., presurgical measurement, within the same group). Results are displayed as median±range.

Mechanical properties of the nerve guides have been evaluated, i.e., for their flexibility and compression resistance. Presurgically, all nerve guides, irrespective of EO or *β* sterilized, showed similar values in their flexibility (EO: 2.4±0.1 mm, *β*: 2.6±0.1 mm; both groups: n=5) and compression resistance (EO: 1.2±0.1 N, *β*: 1.0±0.0 N; both groups: n=5, Figure 6). As earlier described [7], the flexibility was not evaluable after in vivo application of the CNGs. Over time after surgery, a continuously but only slightly higher force was required to compress EO tubes by the same degree as presurgically, whereas *β* sterilized NCGs showed a significant increase in their stiffness over time (much higher force needed to compress these tubes by 60%). Dimensional changes (length, diameter) of the nerve guides have not been observed.

The macroscopically evident degree of degradation for the explanted NCGs was also assessed and again similar for both groups. Earliest evidence of material degradation was found not before 56 weeks after surgery and displayed in macroscopically visible marks of degradation, e.g., fissures or fragmentation, in one tube per group (both groups: n=6). At 76 weeks after surgery, 1 EO-CNG (n=3) and 3 *β*-CNGs (n=6) displayed the same signs of degradation. Furthermore, chitosan fragments were detected in HE-stained crosssections of the surrounding connective tissues in a similar low number of specimen from both groups (56 weeks: EO: in 2 of 6 samples, *β*: in 1 of 6 samples; 76 weeks after surgery: EO: in 1 of 3 samples, *β*: in 1 of 6 samples).

## 4. Discussion

Since beta irradiation (*β*) was poorly studied so far with regard to its influences on the mechanical properties of chitosan implant materials [14, 17], we performed a comprehensive long-term evaluation on this topic in direct comparison to the commonly applied sterilization method in this field, i.e., ethylene oxide gassing (EO) [13–15]. Although we found an increased overall recovery rate of motor function in the *β* group, no significant differences were detected between both methods. In the herein studied Lewis rats, the observed recovery rates of 41.7% (EO group) and 50.0% (*β* group) at 16 weeks after surgery following the reconstruction of 12 mm nerve gaps are in line with our previous studies [4, 6, 7]. In these studies, a recovery rate of 68.8% at 12 weeks after surgery was detected upon 10 mm nerve gap repair using *β*-sterilized CNGs in Wistar rats [6] and a recovery rate of 42.9% at 16 weeks after surgery following 15 mm nerve gap repair using either *β*-sterilized CNGs in Wistar rats [4] or EO-treated CNGs in Lewis rats [7]. The seemingly increase in functional motor recovery observed within the next 60 weeks in the current study is related to the fact that we have always chosen 6 animals per group for interim histological and mechanical evaluation, which previously presented with the worst motor functional recovery data at the respective time points. We have chosen this approach, because we primarily aimed to study the materials' long-term effects at the implantation site and the particular CNG properties. Thereby, we also reduced the period the animals had to suffer from nonfunctional nerves.

With regard to a potential inflammatory tissue reaction related to the material degradation over time and correlated to the selected sterilization method, our long-term study demonstrated a good biocompatibility of the CNGs over 76 weeks in vivo. This time period is much longer than usually studied in the context of peripheral nerve regeneration [26] and almost the longest period to be studied in the rat since we already lost a few animals due to senile decay. We histologically analyzed the connective tissue that had formed around the CNGs with regard to an unusual large tissue area or thickness and the presence of activated macrophages as parameters related to a foreign body reaction. Although *β*-CNGs showed a significantly increased connective tissue thickness by the end of the study, the other parameters did not display significant differences between the groups. And the final tissue thickness values were still within a normal range compared to earlier studies where no immunoreaction was detectable [6, 7]. Furthermore, the number of activated macrophages remained at a constantly low level (range: 5.3-62.3 ED1^+^ cells/mm^2^) compared to our own previous results (*β*-CNGs: 750 ED1^+^ cells/mm^2^ at 18 days after surgery and approximately 200 ED1^+^ cells/mm^2^ at 13 weeks after surgery within the same study) [6]. Overall, the current long-term study did not provide any hints of an ongoing immunoreaction in neither *β*- CNGs nor EO-CNGs.

Characterizing the regrowth of myelinated axons into the distal nerve in correlation with the progressing functional motor recovery, we detected a trend to a better outcome for implantation of *β*-CNGs (number of regrown axons and nerve fiber density), but again without significant differences to implantation of EO-CNGs. In both groups, axonal size parameters remained below the level of healthy nerve samples as expected for regenerated nerves [26].

Evaluating the CNGs mechanical properties presurgically and at different time points up to 76 weeks after implantation, both, *β*-CNGs and EO-CNGs, displayed similar presurgical compression force resistances. The values remained stable for EO-CNGs over time, while we detected a significant increase in material stiffness for *β*-CNGs on the long-term.

With regard to long-term material degradation times, literature claims that beta irradiation of collagen leads to changes in the degradation time due to chain scissions [14], and we therefore expected an earlier degradation of *β*-CNGs. Our study, however, was not designed as detailed degradation study. Nevertheless, first macroscopic signs of degradation could be reported at 56 weeks postimplantation while none of the nerve guides were considerably degraded at the end of the study at 76 weeks. Interestingly, both groups presented a similar progression of degradation over time, despite the potential of chain scissions due to *β*-sterilization [27]. Depending on the dose of irradiation, a faster degradation has been found in experiments with collagen. However, irradiation can also lead to material crosslinking hampering the degradation process [14]. EO sterilization, on the other side, does not lead to polymer degradation or crosslinking reactions, and the chemical structure of chitosan is essentially preserved [15, 27].

In conclusion, our study focused on the comparison of two relevant sterilization methods for nerve guides, EO and *β*-sterilization, in terms of (1) the long-term nerve regeneration and (2) the mechanical strength of the nerve guides, which is an important parameter to support the regrowth and maturation of functional nerve. We found no significant differences between the commonly applied EO and the rarely described *β* sterilization of chitosan materials in our long-term in vivo study. We also did not detect any negative effects that could be related to toxic residues barring in and slowly releasing from EO-CNGs. It could, however, be considered that nerve guide stiffness increased over time for *β*-CNGs, an event that may counteract the efforts to provide highly bendable and still collapse stable nerve guides [7]. Therefore, although functional motor and axonal recovery was minimally increased in the current study after implantation of *β*-CNGs versus EO-CNGs, EO gassing can be considered the more advantageous sterilization method over *β* irradiation.

## Figures and Tables

**Table 1:** Timeline of motor recovery based on evocable compound muscle action potentials (CMAPs) recorded from the anterior tibial (TA) and plantar muscles (PL) upon transcutaneous stimulation of the reconstructed sciatic nerve. Every 20 weeks beginning at 16 weeks after surgery, group sizes were reduced by six animals for interim histomorphometrical analysis. In the EO group, three other animals died due to age in the course of the study (respective n-counts are displayed in the table). A chi-square test was used for statistical analysis of the results (level of significance: p<0.05).

		**16 weeks **		**36 weeks **		**56 weeks **		**76 weeks **	
		**after surgery**		**after surgery**		**after surgery**		**after surgery**	
**TA**	**EO**	9/24	37.5%	11/18	61.1%	9/11	81.8%	3/3	100.0%
	***β***	12/24	50.0%	12/18	66.7%	11/12	91.7%	6/6	100.0%

**PL**	**EO**	10/24	41.7%	10/18	55.6%	7/11	63.6%	3/3	100.0%
	***β***	12/24	50.0%	11/18	61.1%	10/12	83.3%	6/6	100.0%

## Data Availability

The datasets analyzed during this study are available from the corresponding author on request. Raw data are stored in the authors' institutional repositories and will be accordingly provided upon request.
